# Foot-and-mouth disease virus replicates independently of phosphatidylinositol 4-phosphate and type III phosphatidylinositol 4-kinases

**DOI:** 10.1099/jgv.0.000485

**Published:** 2016-08

**Authors:** Stephen Berryman, Katy Moffat, Christian Harak, Volker Lohmann, Terry Jackson

**Affiliations:** ^1^​ The Pirbright Institute, Ash Rd, Pirbright, Surrey, GU24 0NF, UK; ^2^​ Department of Infectious Diseases, Molecular Virology, University of Heidelberg, Heidelberg, Germany

**Keywords:** PI4P, PI4KIIIα, PI4KIIIβ, cholesterol, FMDV, replication

## Abstract

Picornaviruses form replication complexes in association with membranes in structures called replication organelles. Common themes to emerge from studies of picornavirus replication are the need for cholesterol and phosphatidylinositol 4-phosphate (PI4P). In infected cells, type III phosphatidylinositol 4-kinases (PI4KIIIs) generate elevated levels of PI4P, which is then exchanged for cholesterol at replication organelles. For the enteroviruses, replication organelles form at Golgi membranes in a process that utilizes PI4KIIIβ. Other picornaviruses, for example the cardioviruses, are believed to initiate replication at the endoplasmic reticulum and subvert PI4KIIIα to generate PI4P. Here we investigated the role of PI4KIII in foot-and-mouth disease virus (FMDV) replication. Our results showed that, in contrast to the enteroviruses and the cardioviruses, FMDV replication does not require PI4KIII (PI4KIIIα and PI4KIIIβ), and PI4P levels do not increase in FMDV-infected cells and PI4P is not seen at replication organelles. These results point to a unique requirement towards lipids at the FMDV replication membranes.

## Introduction

The family *Picornaviridae* belongs to the order *Picornavirales *and currently consists of 50 species grouped into 29 genera that include many significant pathogens of man and animals such as poliovirus (PV), human rhinoviruses (HRV), enterovirus 71 (EV71), coxsackieviruses (CV), hepatitis A virus (HAV) and foot-and-mouth disease virus (FMDV). On infection, picornaviruses induce formation of new membrane structures, termed replication organelles (RO) that are specialist sites dedicated to virus replication (Belov, 2014; Belov & Sztul, 2014; Belov & van Kuppeveld, 2012). Over recent years, accumulative evidence has suggested that picornaviruses form RO by remodelling membranes of the early secretory pathway; however, the precise origin of the membranes is not clear and may differ for different virus genera. For example, enteroviruses (PV, CV and HRV) initiate formation of RO at Golgi membranes (Belov, 2014; Belov & Sztul, 2014; Hsu *et al.*, 2010) while replication of the cardioviruses [encephalomyocarditis virus (EMCV)] is believed to start at the endoplasmic reticulum (ER) (Gazina *et al.*, 2002). Furthermore, the mechanisms that generate RO appear to differ for the different genera, and a number of cellular proteins including PI4K (phosphatidylinositol 4-kinase), Arf1 (ADP-ribosylation factor 1), GBF1 (Golgi brefeldin A resistant guanine nucleotide exchange factor 1), Sar1 (secretion-associated and Ras-related 1), ACBD3 [acyl-coenzyme A (CoA)-binding protein domain 3] and OSBP (oxysterol-binding protein) have been implicated in facilitating replication of different viruses (Arita, 2014; Belov *et al.*, 2008; Greninger *et al.*, 2012; Hsu *et al.*, 2010; Ishikawa-Sasaki *et al.*, 2014; Lanke *et al.*, 2009; Midgley *et al.*, 2013; Roulin *et al.*, 2014; Sasaki *et al.*, 2012; Strating *et al.*, 2015; Wessels *et al.*, 2006). Despite these differences, a common theme emerging from studies investigating picornavirus replication is the requirement for phosphatidylinositol 4-phosphate (PI4P), and, for a number of picornaviruses, PI4P levels are increased in infected cells and PI4P has been shown to localize to RO (Delang *et al.*, 2012; Dorobantu *et al.*, 2015; Greninger *et al.*, 2012; Roulin *et al.*, 2014; Sasaki *et al.*, 2012; Spickler *et al.*, 2013; Strating *et al.*, 2015). At RO, PI4P is believed to facilitate recruitment of viral proteins, such as the viral RNA-dependent RNA-polymerase (known as 3D) (Hsu *et al.*, 2010), and cellular proteins that support virus replication, such as OSBP (Arita, 2014; Roulin *et al.*, 2014; Strating *et al.*, 2015). OSBP plays a central role in cholesterol transport and shuttles cholesterol between different cellular organelles in exchange for PI4P (Mesmin *et al.*, 2013). Enteroviruses and cardioviruses have been shown to subvert OSBP to exchange PI4P for cholesterol at RO (Albulescu *et al.*, 2015b; Arita *et al.*, 2013; Dorobantu *et al.*, 2015; Ilnytska *et al.*, 2013; Roulin *et al.*, 2014; Strating *et al.*, 2015; van der Schaar *et al.*, 2013). Consequently, inhibition or depletion of OSBP inhibits replication of enteroviruses and cardioviruses; however, OSBP is not required by all picornaviruses and infections by equine rhinitis A virus (ERAV; genus *Aphthovirus*) and human parechoviruses (genus *Parechovirus*) are OSBP independent (Strating *et al.*, 2015).

PI4P is generated at RO through the activity of lipid kinases. PI4KIIIβ is localized at RO in enterovirus-infected cells, and inhibition or depletion of PI4KIIIβ inhibits replication (Dorobantu *et al.*, 2015; Greninger *et al.*, 2012; Roulin *et al.*, 2014; Sasaki *et al.*, 2012; Spickler *et al.*, 2013; Strating *et al.*, 2015; van der Schaar *et al.*, 2013; Wessels *et al.*, 2006). In contrast, EMCV has been shown not to require PI4KIIIβ, instead relying on PI4KIIIα to generate PI4P at RO (Dorobantu *et al.*, 2015). The requirement for different isoforms of PI4K may reflect the differential use of ER and Golgi membranes as the initial replication site, as PI4KIIIα is predominantly located on the ER whereas PI4KIIIβ is enriched on the Golgi (Balla, 2013; Godi *et al.*, 1999). In addition, replication of certain HRV appears to require both PI4KIIIβ and PI4KIIIα (Roulin *et al.*, 2014).

FMDV is the prototype species of the *Aphthovirus* genus of the *Picornaviridae* and the causative agent of foot-and-mouth disease (FMD), which affects cattle, sheep, goats and pigs. FMD is endemic in many regions of the world and causes enormous economic losses to livestock industries (Knight-Jones & Rushton, 2013). Similarly to other picornaviruses, electron microscopy studies have shown that FMDV infection of (BHK) cells (Monaghan *et al.*, 2004) and IBRS2 cells (Martin-Acebes *et al.*, 2008) generates large numbers of single- and double-membrane vesicles, which are likely RO. The ER-Golgi intermediate compartment (ERGIC) and Golgi are disrupted early after infection and before major changes are detected in the ER (Midgley *et al.*, 2013; Zhou *et al.*, 2013). However, replication of FMDV appears to differ from that of the enteroviruses in a number of ways; for example, enterovirus replication requires an intact Golgi and is inhibited by Golgi disruption caused by inhibition of Arf1 or by brefeldin A (BFA) (Cuconati *et al.*, 1998; Doedens *et al.*, 1994; Gazina *et al.*, 2002; Irurzun *et al.*, 1992; Lanke *et al.*, 2009; Maynell *et al.*, 1992). In contrast, FMDV (along with the cardioviruses) is unusual among the picornaviridae by being resistant to BFA, and infection does not require an intact Golgi (Gazina *et al.*, 2002; Martin-Acebes *et al.*, 2008; Midgley *et al.*, 2013; Monaghan *et al.*, 2004; O'Donnell *et al.*, 2001). Furthermore, we and others have shown that Golgi disruption appears to make FMDV replication more favourable as infection is enhanced by disruption resulting from either expression of dominant negative Arf1 or by BFA (Martin-Acebes *et al.*, 2008; Midgley *et al.*, 2013). These observations suggest that if FMDV requires membranes of the early secretory pathway to form RO they likely derive from pre-Golgi membranes such as the ER or ERGIC. The ER comes in to close apposition with other organelles, including the Golgi, at membrane contact sites (reviewed by Phillips & Voeltz, 2016). Lipid exchange at ER-Golgi contact sites by transport proteins such as OSBP is important for intracellular lipid homeostasis (Mesmin *et al.*, 2013). As described above, this process has been hijacked by enteroviruses which utilize PI4K to synthesize PI4P at RO, which is then exchanged for cholesterol by OSBP, thus driving accumulation of cholesterol at RO (Roulin *et al.*, 2014).

Here, we have investigated the role of PI4P and PI4K in FMDV replication. Our results show that, in contrast to the enteroviruses, cardioviruses and kobuviruses, PI4P levels do not increase in FMDV-infected cells and PI4P is not present at RO. Consistent with these observations, we also show that FMDV infection does not require PI4KIIIα or PI4KIIIβ. These results point to a unique requirement towards lipids at the FMDV replication membranes.

## Results

### Investigating components of FMDV RO

**Fig. 1.**
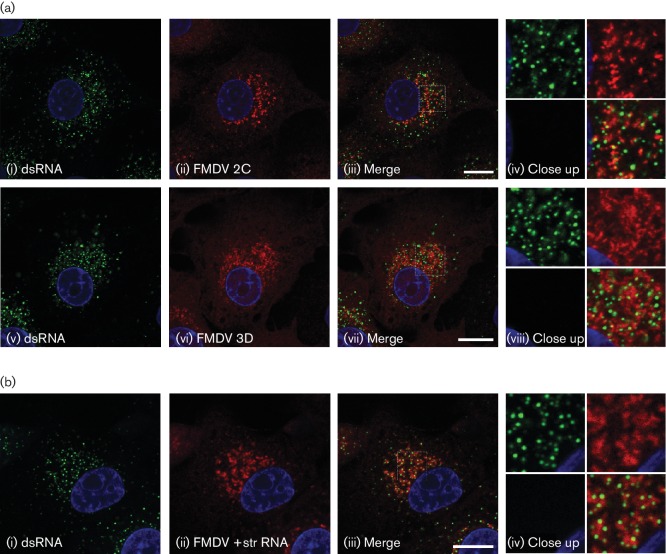
IBRS2 cells were infected with FMDV (m.o.i.=0.8) for 3 h 45 min, fixed and processed for confocal microscopy. Nuclei are blue. Bars, 10 µm. (a) Cells were double-labelled for dsRNA (green, i and v) and for FMDV 2C or 3D (ii and vi, respectively, red). (b) Cells were double-labelled for dsRNA (green, i) and FMDV positive-sense RNA (red, ii) by *in situ* hybridization.

**Fig. 2.**
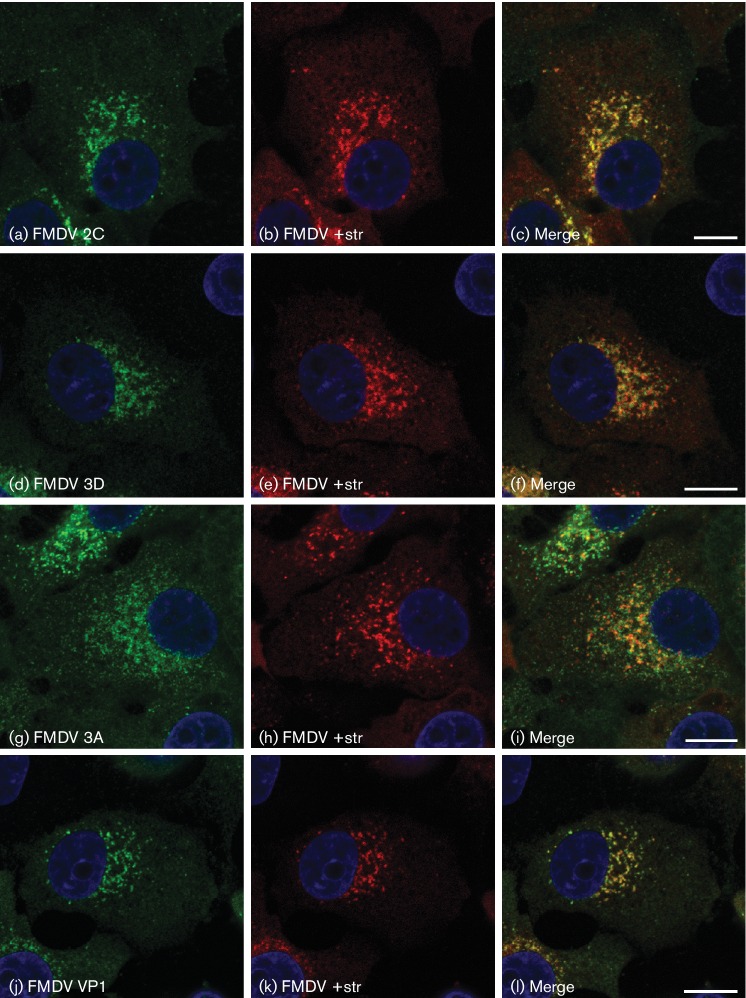
IBRS2 cells were infected with FMDV (m.o.i.=0.8) for 3 h 45 min, fixed and processed for confocal microscopy. Nuclei are blue. Bars, 10 µm. Cells were double-labelled for FMDV positive-sense RNA (red, b, e, h, k) by *in situ* hybridization, and for FMDV 2C, 3D, 3A or VP1 (green, a, d, g, j, respectively).

There is little information describing formation of RO in FMDV-infected cells (Knox *et al.*, 2005; Martin-Acebes *et al.*, 2008; Midgley *et al.*, 2013; Monaghan *et al.*, 2004). Therefore, our initial studies focused on identifying the viral proteins and host factors present at RO. For these studies we used IBRS2 cells. FMDV replication in IBRS2 cells is relatively fast, and infection results in cell death by 5–6 h. Therefore, we investigated RO at 3.5–4.0 h post-infection (p.i.), when replication has been shown to be maximal for IBRS2 cells (Midgley *et al.*, 2013; Rodriguez Pulido *et al.*, 2009). Consistent with these observations, at this time post-infection, labelling for dsRNA (which is produced during infection; Agol *et al.*, 1999) is greatly enhanced in FMDV-infected cells (Fig. S1, available in the online Supplementary Material), as detected using mAb J2 (which recognizes lengths of dsRNA ≥40 bp). Fig. 1(a) shows infected cells co-labelled for dsRNA using mAb J2 and the viral non-structural proteins (nsp), 2C and 3D. These proteins are established markers of RO and might be expected to co-localize with dsRNA; however, for both proteins, little or no co-localization with dsRNA was observed. Fig. 1(b) also shows labelling for dsRNA combined with *in-situ* hybridization for positive-strand viral RNA. Again, although they appeared in close proximity, little co-localization was seen. To investigate this further, we determined co-localization of viral nsp with positive-strand RNA and observed extensive co-localization of positive-strand RNA with 2C and 3D (Fig. 2). The 3A protein was also co-localized with positive-strand RNA; however, significant 3A labelling was also seen distinct from the positive-strand signal. In addition, positive-strand RNA was extensively co-localized with the capsid protein VP1. These results show that in FMDV-infected cells, dsRNA is not localized with accepted markers of RO (i.e. viral nsp) or with positive-strand RNA. Furthermore, positive-strand RNA shows extensive co-localization with both nsp and capsid proteins suggesting that in FMDV-infected cells, dsRNA and other viral components are spatially segregated at RO.

**Fig. 3.**
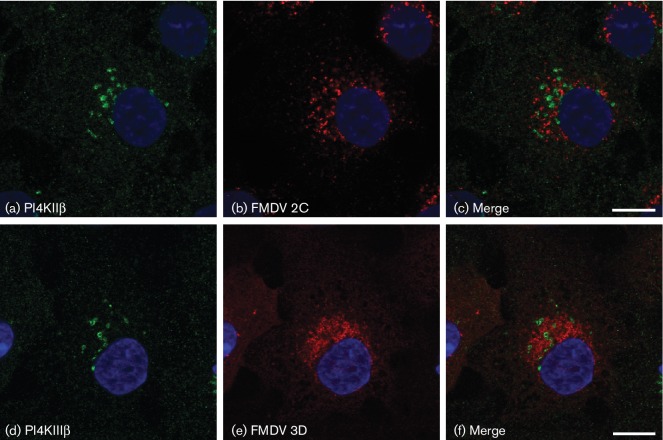
IBRS2 cells were infected with FMDV (m.o.i.=0.8) for 3 h 45 min, fixed and processed for confocal microscopy. Nuclei are blue. Bars, 10 µm. Cells were double-labelled for PI4KIIIβ (green, a and d) and for FMDV 2C or 3D (red, b and e, respectively).

**Fig. 4.**
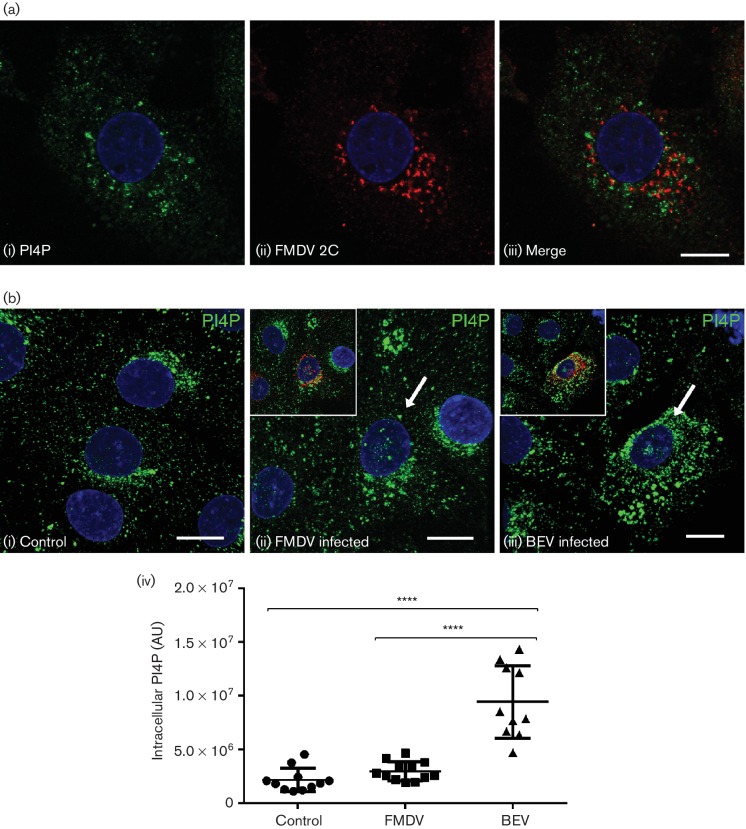
IBRS2 cells were infected with FMDV or BEV-1 (m.o.i.=0.5) for 3 h 30 min, fixed and processed for confocal microscopy. Cells were labelled for intracellular PI4P using a PI4P-specific antibody following permeabilization with digitonin. Nuclei are blue. Bars, 10 µm. (a) FMDV-infected cells were double-labelled for intracellular PI4P (green, i) and for FMDV 2C protein (red, ii). Images shown are single optical sections through the middle of the cell. (b) Intracellular PI4P was quantified by immunofluorescence analysis. Mock-infected, FMDV-infected or BEV-infected IBRS2 cells (m.o.i.=0.6) were labelled for intracellular PI4P. FMDV-infected cells were identified by labelling for FMDV 3A, and BEV-infected cells were identified by labelling using a guinea pig antiserum raised against whole virus. Example images taken from mock-infected (i), FMDV-infected (ii) and BEV-infected samples (iii) are shown as maximum projections of z-stacks, with PI4P in green and infected cells distinguished by white arrows and by red labelling in insets. (iv) Intensities of intracellular fluorescent PI4P signals from whole-cell z-stacks (spacing 0.3 µm) were quantified from at least 10 cells per condition using Imaris image analysis software. Error bars show standard deviation. *P* values were calculated using one-way ANOVA. Significant difference (95 % confidence level using TUKEY-style multiple comparisons test) was found between FMDV and BEV (****P<0.0001), and control and BEV (****P<0.0001). There was no significant difference between FMDV and the control (not significant *P*>0.05). AU, Arbitrary units.

PI4KIIIβ is required for enterovirus replication and has been shown to co-localize at RO with viral nsp such as 3A and 3D (see Introduction). In IBRS2 cells, labelling for PI4KIIIβ showed the expected peri-nuclear pattern consistent with a Golgi localization (data not shown). However, in contrast to enteroviruses, although labelling was dispersed in FMDV-infected cells, co-localization of PI4KIIIβ at RO with nsp (2C or 3D) was not seen (Fig. 3). As a result of PI4KIIIβ activity in enterovirus-infected cells, PI4P levels are increased and PI4P is also present at RO (Hsu *et al.*, 2010). Similar to PI4KIIIβ, labelling for PI4P was predominantly peri-nuclear in uninfected IBRS2 cells and dispersed by FMDV infection. Furthermore, PI4P was not co-localized at RO with the viral 2C protein (Fig. 4a). Similar observations were made for dsRNA, as co-localization with PI4P was not seen in infected cells (Fig. S2). Fig. 4(b) shows an analysis of intracellular PI4P levels for cells infected with FMDV or bovine enterovirus (BEV). Consistent with previous reports for enteroviruses, in BEV-infected cells PI4P was dispersed and PI4P levels were found to be elevated. However, in contrast to BEV, although PI4P was dispersed, PI4P levels did not appear altered by FMDV infection (Fig. 4b). Together, these results show that intracellular PI4P levels are not increased in FMDV-infected cells, and PI4KIIIβ and PI4P are not associated with RO suggesting that, unlike the enteroviruses, FMDV does not require PI4KIIIβ or PI4P for infection.

### Investigating the role of PI4K in FMDV infection

**Fig. 5.**
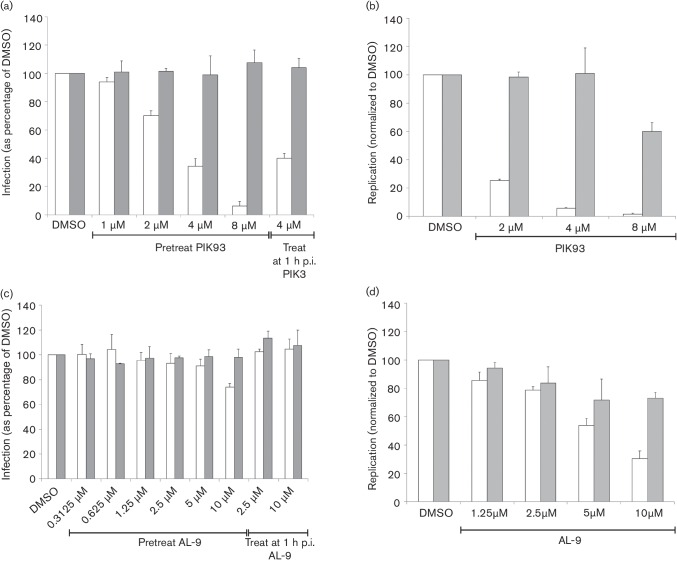
(a, c) IBRS2 cells in 96-well plates were pre-treated for 1 h with PIK93 (a) or AL-9 (c) at the concentrations indicated (or DMSO as a control) and then infected with FMDV (grey bars) or BEV-1 (white bars) at (m.o.i.=0.3) in the presence of drug for 1 h. Virus was washed away, and then infection continued for a further 3 h (FMDV) or 4 h (BEV-1). Infection was stopped, the cells were fixed and permeabilized, and infected cells were identified as described in Methods. The results were normalized to the DMSO-treated controls. Shown are the means±sd for triplicate wells. Each panel shows one experiment representative of two conducted, each giving similar results. (b, d) IBRS2 cells in white-walled 96-well plates were transfected with FMDV (grey bars) or CVB3 (white bars) renilla luciferase replicon RNA and incubated at 37 °C in the presence of PIK93 (b) or AL-9 (d) at the concentrations indicated (or DMSO as a control) and Enduren renilla luciferase substrate. Luminescence was measured after 5 h (FMDV replicon) or 6 h (CVB3 replicon). The results were normalized to the DMSO-treated controls. Shown are the means±sd for triplicate wells. Each panel shows one experiment representative of two conducted, each giving similar results.

To further investigate the role for PI4K in FMDV infection we used PIK93, which inhibits PI4KIIIβ (Balla *et al.*, 2008). For this study we included BEV, as enteroviruses require PI4KIIIβ for replication and infection is inhibited by PIK93 (Roulin *et al.*, 2014; van der Schaar *et al.*, 2013). Consistent with observations with other enteroviruses, PIK93 had a potent inhibitory effect on BEV infection (Fig. 5a), including when added 1 h after infection. In contrast, PIK93 did not inhibit FMDV infection. We also compared the effect of PIK93 on FMDV replication using a luciferase-based sub-genomic replicon (Fig. 5b). For comparison, we included a Coxsackievirus B3 (CVB3) replicon (Lanke *et al.*, 2009) as CVB3 requires PI4KIIIβ for replication and is inhibited by PIK93 (Hsu *et al.*, 2010; van der Schaar *et al.*, 2013). PIK93 inhibited CVB3 replication by ~90 % at 4 µM, while FMDV replication was not inhibited at this concentration. However, PIK93 also inhibited FMDV replication, but to a lesser extent and only by ~40 % at 8 µM. At higher concentrations, PIK93 is known to also inhibit PI4KIIIα (Balla *et al.*, 2008), and the reduced luciferase signal from the FMDV replicon caused by higher concentrations of PIK93 could indicate a role for PI4KIIIα in replication. Therefore, we also investigated if FMDV infection requires PI4KIIIα using AL-9, which preferentially targets PI4KIIIα (Bianco *et al.*, 2012). This analysis showed that at 10 µM, AL-9 had a small inhibitory effect on BEV infection but not on FMDV (Fig. 5c). Similarly, at 10 µM AL-9 had a small inhibitory effect on the FMDV (~25 %) replicon (Fig. 5d). At this concentration, AL-9 also inhibited the CVB3 replicon by ~65 %. However, although at low concentrations AL-9 preferentially targets PI4KIIIα, at 10 µM, AL-9 is reported to also inhibit PI4KIIIβ (Bianco *et al.*, 2012) suggesting that the effect on CVB3 may be mediated through inhibition of PI4KIIIβ. This conclusion is supported by the observations that hepatitis C virus replication requires PI4KIIIα and is inhibited by much lower concentrations of AL-9 (IC_50_<1 uM) (Bianco *et al.*, 2012). Together, the above observations suggest that the small inhibitory effects of PIK93 on FMDV infection/replication more likely result from non-specific, off-target effects and support the conclusion that FMDV does not require PI4KIIIβ or PI4P for replication. Furthermore, our results suggest that FMDV replication is also independent of PI4KIIIα.

**Fig. 6.**
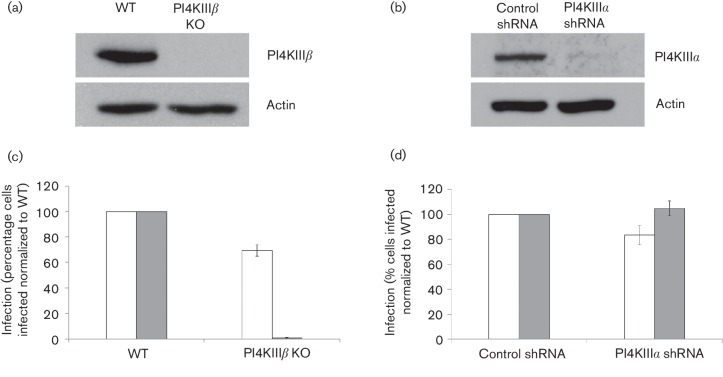
Western blot for (a) PI4KIIIβ in WT and PI4KIIIβ knockout (PI4KIIIβ KO) Huh7-Lunet T7 cells (8 µg protein loaded per lane) and (b) PI4KIIIα in Huh7-Lunet T7 cells expressing a control or PI4KIIIα shRNA (18 µg protein loaded per lane). Actin was included as a loading control. (c) WT (control) and PI4KIIIβ KO Huh7-Lunet T7 cells and (d) Huh7-Lunet T7 cells expressing a control shRNA (control) or PI4KIIIα shRNA were grown on glass coverslips infected with FMDV (white bars) and BEV-1 (grey bars) (m.o.i.=0.6) for 4 h, fixed and processed for confocal microscopy. Infected cells were identified by labelling for FMDV 3A or BEV-1, respectively. Infection was scored and normalized to that seen in control cells for each virus. The mean±sd for triplicate coverslips are shown (*n*>650 per coverslip).

To further investigate the role of PI4KIIIα and PI4KIIIβ in FMDV infection we used Huh7 cell lines where levels of PI4KIIIα were reduced by small hairpin RNA (shRNA) (Reiss *et al.*, 2013), or expression of PI4KIIIβ inhibited by gene knockout (Esser-Nobis *et al.*, 2015). Western blot analysis confirmed the reduced levels of PI4KIIIα and PI4KIIIβ in these cell lines (Fig. 6a, b). Next, we infected these cells with either FMDV or BEV and processed samples for confocal microscopy, labelling for viral proteins. For each matched pair of cells (parental and PI4K-depleted), the proportion of cells infected was determined and normalized to the parental cells. This analysis showed that, consistent with previous findings that show enteroviruses require PI4KIIIβ for replication, the PI4KIIIβ knockout cells were infected poorly by BEV (Fig. 6c). In contrast, infection by FMDV was reduced by only ~30 % confirming that FMDV does not require PI4KIIIβ for infection (Fig. 6c). Fig. 6 also shows that reduced expression of PI4KIIIα did not affect infection by either FMDV or BEV (Fig. 6d). Together, these results confirm that FMDV does not require the catalytic activity of PI4KIIIα or PI4KIIIβ for infection, and demonstrate additionally that infection does not require any non-enzymic activities of these proteins.

## Discussion

To date, the best-studied picornaviruses are the enteroviruses and they have been invaluable as models to study picornavirus replication. However, the *Picornaviridae* is now recognized as one of the largest and most diversified virus families, and since 2012 the number of genera has increased from 12 (including 28 species) to the current 29, including at least 50 species (Knowles *et al.*, 2012). It is now clear that, although picornaviruses share a common replication strategy, different host factors and cellular pathways contribute to the replication of different genera. Despite these differences, common themes to emerge from studies of RO formation are the requirements for PI4K, PI4P and cholesterol. For example, in enterovirus-infected cells, recruitment of PI4KIIIβ leads to the local production of PI4P at RO and the subsequent recruitment of viral (e.g. 3D) and host proteins (e.g. OSBP) that participate in viral genome replication (see Introduction). In addition, kobuviruses and cardioviruses have recently been shown to require PI4K (PI4KIIIβ and PI4KIIIα, respectively) for replication (Dorobantu *et al.*, 2015; Greninger *et al.*, 2012; Sasaki *et al.*, 2012), which likely generate PI4P at RO. Here we have shown that FMDV appears to use a novel replication strategy that does not require PI4KIIIα or PI4KIIIβ. Furthermore, we have shown that intracellular levels of PI4P were not elevated in FMDV-infected cells and PI4P is not localized at RO. Recently, HAV was also shown to replicate independently from PI4KIIIα or PI4KIIIβ (Esser-Nobis *et al.*, 2015) suggesting that picornaviruses from diverse genera (FMDV and HAV) have evolved to replicate independently from type III PI4Ks.

The observation that FMDV does not require PI4KIIIα is consistent with a previous report that showed that ERAV infection was not inhibited by AL-9 (Dorobantu *et al.*, 2015). It is possible that FMDV could use either PI4KIIα or PI4KIIβ in place of type III PI4Ks used by other picornaviruses; however, this is unlikely since we have also shown that levels of intracellular PI4P were not elevated in FMDV-infected cells and PI4P is not localized at RO. For the enteroviruses and cardioviruses, the viral 3A protein has a key role in formation of the RO and recruits PI4K (Hsu *et al.*, 2010), and 3A of PV, HRV and CVB3 have been shown to recruit PI4KIIIβ to RO, while the 3A of EMCV recruits PI4KIIIα (Dorobantu *et al.*, 2015; Greninger *et al.*, 2012). Interestingly, a single point mutation in 3A of CVB3 confers resistance to drugs targeting PI4KIIIβ and also enables the virus to replicate independently of PI4KIIIβ and raised levels of PI4P on internal membranes, which are required by the parental virus (van der Schaar *et al.*, 2012). Thus, these drug-resistant variants of CVB3 behave similarly to FMDV in that they demonstrate the ability to replicate independently of PI4K activity or elevated intracellular levels of PI4P. The FMDV 3A protein is much longer than that of enteroviruses and cardioviruses and appears functionally distinct as the enterovirus 3A protein inhibits protein secretion, whereas for FMDV, secretion is not blocked by 3A but instead by 2B and 2C (Moffat *et al.*, 2005, 2007). Given these differences, it is interesting to speculate that FMDV 3A may recruit alternative host factors that enable virus replication independently of PI4K and PI4P.

Our results show that the membranes of FMDV RO lack PI4P and therefore have a different lipid composition to those found in enterovirus-infected cells. In enterovirus-infected cells PI4P is exchanged for cholesterol at RO by OSBP, and inhibition of OSBP by 25-hydroxycholesterol (25-HC) inhibits virus replication (Arita *et al.*, 2013; Civra *et al.*, 2014; Roulin *et al.*, 2014). 25-HC targets cholesterol synthesis in addition to cholesterol/PI4P exchange by OSBP; however, enterovirus replication is unaffected by other cholesterol synthesis inhibitors such as AY9944 and compactin (Roulin *et al.*, 2014), whilst remaining sensitive to the OSBP inhibitor OSW-1 (Albulescu *et al.*, 2015a) and knockdown of OSBP by siRNA (small interfering RNA) (Roulin *et al.*, 2014; Strating *et al.*, 2015). This indicates that the effect of 25-HC on enterovirus replication is mediated by its impact on OSBP activity. Interestingly, our preliminary data suggests that FMDV is less sensitive than BEV to 25-HC (data not shown), and therefore may not require OSBP for replication. These observations are consistent with previous studies showing that ERAV (also genus *Aphthovirus*) replicates independently of OSBP and provides further evidence that aphthovirus RO form by different mechanisms to those of enteroviruses. However, further study will be required to determine the role of OSBP and cholesterol in FMDV replication if any.

Our results show that in FMDV-infected cells, positive-strand vRNA localized with 3A, 2C and 3D, which are accepted markers of picornavirus RO. However, neither positive-strand vRNA nor nsp was co-localized with dsRNA. This is a little surprising as dsRNA and the nsp might be expected to be present at RO. In contrast to our results, it has been reported that dsRNA is co-localized with viral nsp in Aichi virus- (Sasaki *et al.*, 2012) and poliovirus-infected cells (Richards *et al.*, 2014), and with PI4KIIIβ in HRV- and echovirus-infected cells (Ilnytska *et al.*, 2013). However, although in HRV-infected cells most PI4KIIIβ was in close proximity with dsRNA, some dsRNA labelling appeared not to be associated with PI4KIIIβ (Ilnytska *et al.*, 2013). In addition, more recently, in cells infected with EMCV, dsRNA was shown to be in close association but not extensively overlapping with PI4KIIIα, a marker of RO that co-localizes extensively with EMCV 3AB (Dorobantu *et al.*, 2015). This last report closely supports our data in suggesting that for some picornaviruses, including EMCV and FMDV, dsRNA labelling is spatially segregated from viral replication proteins as shown previously for coronaviruses (Hagemeijer *et al.*, 2012; Knoops *et al.*, 2008).

In summary, our data show that FMDV uses a novel approach to generate RO. In contrast to enteroviruses and cardioviruses, FMDV does not require PI4KIIIα or PI4KIIIβ for infection and PI4P is not elevated or localized to RO in infected cells, demonstrating the possibility of a variety of different RO lipid compositions in the family *Picornaviridae*.

## Methods

### Cells and viruses

IBRS2 cells were cultivated in Glasgow’s modified Eagle’s medium (GMEM - obtained from Sigma) with 10 % adult bovine serum, BHK cells in GMEM with 10 % FCS and 5 % tryptose phosphate broth, and Huh7 cell lines in Dulbecco’s modified Eagle’s medium (Sigma) with 10 % FCS. All media were supplemented with 20 mM l-glutamine, 100 SI units penicillin ml^−1^ and 100 µg streptomycin ml^−1^ (All from Sigma). Huh7-Lunet T7 cells expressing control shRNA or PI4KIIIα shRNA (Reiss *et al.*, 2013) were grown in media supplemented additionally with 5 µg zeocin ml^−1^and 2 µg puromycin ml^−1^ (both Life Technologies). Huh7-Lunet T7 cells and PI4KIIIβ knockout Huh7-Lunet T7 cells (Esser-Nobis *et al.*, 2015) were grown in media supplemented additionally with 5 µg zeocin ml^−1^ only. Working stocks of FMDV O1K were prepared using BHK cells as described previously (Berryman *et al.*, 2005; Jackson *et al.*, 1996). Working stocks of BEV-1 were prepared using IBRS2 cells. The m.o.i. was based on the virus titre on IBRS2 cells, as described previously (Jackson *et al.*, 2000).

### Antibodies and reagents

Murine mAbs against dsRNA (mAb J2), PI4KIIIβ and PI4P were purchased from English and Scientific Consulting, BD Transduction Laboratories, and Echelon biosciences, respectively. Rabbit antibody against PI4KIIIα for Western blotting was obtained from Cell Signalling Technology. The murine mAb against actin was from Sigma. The mAbs 2C2, 3B5 and D9, which recognize the FMDV 3A, 3D and VP1 proteins (type O viruses), respectively, were gifts from Emiliana Brocchi (IZS, Brescia, Italy). Rabbit antibody against FMDV 2C protein was produced in-house, BEV-infected cells were detected using a guinea pig polyclonal serum generated using whole virus as the immunogen (Midgley *et al.*, 2013). Alexa-Fluor-conjugated secondary antibodies were from Thermo-Fisher. Stock solutions of PIK93 (Merck Millipore) and AL-9 (Petra Neddermann, Istituto Nazionale Genetica Molecolare, Milan, Italy) were prepared in DMSO.

### Immunofluorescence confocal microscopy

#### Infection of cells.

Cells grown on glass coverslips were inoculated with the indicated m.o.i. of FMDV O1K or BEV-1 diluted in cell culture medium containing 1 % FCS (FMDV) or 1 % porcine serum (BEV-1) for 1 h at 37 °C. Unbound virus or capsid was removed by washing, and the cells were returned to 37 °C in cell culture medium with reduced serum for the remainder of the infection time. At the end of the infection time, cells were fixed in 4 % paraformaldehyde for 40 min.

#### Antibody labelling.

Following fixation, cells were permeabilized for 5 min using digitonin (50 µg ml^−1^) when labelling for PI4P, or for 15 min with 0.1 % Triton X-100 in PBS in all other cases, including labelling of dsRNA using mAb J2. Coverslips were then washed, and incubated in block buffer (10 mM Tris, 150 mM NaCl, 1 mM CaCl_2_, 0.5 mM MgCl_2_, 10 %, v/v, normal goat serum and 1 %, v/v, fish skin gelatin) for 0.5 h. Primary antibodies were added for 1 h in block buffer. The cells were washed and incubated with Alexa-Fluor-conjugated secondary antibodies in block buffer for 45 min. After washing, the coverslips were mounted onto microscope slides using Vectashield mounting medium with DAPI (Vector Laboratories).

#### Simultaneous *in situ* hybridization and antibody labelling.

Following fixation, cells were permeabilized for 1 h with 70 % ethanol at 4 °C, and washed with wash buffer (300 mM NaCl, 30 mM sodium citrate dihydrate, 10 %, v/v, formamide). Cells were then incubated for 4 h at 37 °C with hybridization buffer [wash buffer supplemented with 10 %, w/v, dextran sulphate (Mr, 500 000)] containing a Cal-Fluor 590 labelled Stellaris *in situ* hybridization probe set (125 nM) and primary antibody against an antigen of interest. The probe set is designed to recognize positive-sense RNA from FMDV O1K and contains 48 different fluorescently labelled probes designed to hybridize with the non-structural protein region (2B-3D) of the RNA. The coverslips were then twice incubated for 30 min at 37 °C in wash buffer containing secondary antibody, washed once with 2× SSC buffer (300 mM NaCl, 30 mM sodium citrate dihydrate), twice with PBS, and mounted onto microscope slides using Vectashield mounting medium with DAPI.

### Imaging and image analysis

Cells were viewed using a Leica SP2 laser-scanning confocal microscope and optical sections recorded using either the ×63 or ×40 oil-immersion objective with a numerical aperture of 1.4 and 1.25, respectively. The data are shown as single optical sections through the middle of the cell with the exception of Fig. 4(b), which shows maximum projections of z-stacks (spacing 0.3 µm). All data were collected sequentially to minimize cross-talk between fluorescent signals. Images were processed using Adobe Photoshop software.

For quantification of PI4P using Imaris image analysis software (Bitplane Scientific Software), images were recorded in sequential scanning mode. Three-dimensional datasets of cells labelled for PI4P and FMDV 3A, or PI4P and BEV-1 virions, were acquired using the Leica SP2 stack function (spacing 0.3 µm). PI4P labelled structures were detected with the spot function of Imaris. The sum fluorescent intensity for each spot (sum of the intensity of all voxels in the spot) was exported in to Microsoft excel, and the sum PI4P fluorescent intensity was then calculated for each cell.

### FMDV infection assays

#### 96-Well plate assay.

The assay to quantify infection has been described in detail previously (Berryman *et al.*, 2005). Briefly, cells in 96-well tissue culture plates were grown until approximately 90 % confluent. The cells were incubated for 1 h at 37 °C with FMDV or BEV-1 at an m.o.i of 0.3 p.f.u. cell^−1^. The monolayers were washed and incubated with serum-free medium at 37 °C for a further 3 h (FMDV) or 4 h (BEV). Infection was stopped, and the cells were fixed with 4 % paraformaldehyde. The cells were permeabilized with 0.1 % Triton and incubated with blocking buffer [10 mM Tris-HCl (pH 7.5), 140 mM NaCl, 1 mM CaCl_2_, 0.5 mM MgCl_2_, 10 %, v/v, normal goat serum, 1 %, v/v, fish gelatin]. Infected cells were identified by sequential incubation with MAb 2C2 (FMDV) or whole virus antiserum (BEV), biotinylated goat anti-mouse or guinea pig IgG secondary antibody (Southern Biotechnologies) and streptavidin-conjugated alkaline phosphatase (Invitrogen), each for 1 h at room temperature, followed by alkaline phosphatase substrate (Bio-Rad) for 10 min. The cells were then washed with distilled water. Infected cells stained dark blue and were counted using an ELISA plate reader (Zeiss). Inhibitors were added to the cells 1 h prior to the addition of virus and remained present until fixation. Each experiment was carried out with triplicate samples for each condition.

#### Quantification of infection of Huh7 cell lines by confocal microscopy.

Huh7-Lunet T7 cell lines were grown on glass coverslips, infected with BEV or FMDV (m.o.i.=0.6) for 4 h, fixed and processed for confocal microscopy as above, using mAb 2C2 to label FMDV-infected cells or guinea pig polyclonal serum to detect BEV-1-infected cells. The proportion of infected cells was scored and normalized to control cells.

### Replicon assays

An FMDV renilla luciferase replicon was derived from the infectious copy plasmid pT7S3 (Ellard *et al.*, 1999) by replacement of VP4 residue 19 to VP1 residue 213 (encompassing all of capsid proteins VP2 and VP3 and the majority of VP1 and VP4) by renilla luciferase. A CVB3 renilla luciferase replicon (Lanke *et al.*, 2009) was obtained from Frank van Kuppeveld (University of Utrecht, Netherlands). Replicon plasmids were linearized and FMDV and CVB3 replicon RNA was transcribed using the T7 Megascript kit (Ambion) and purified using the Megaclear Kit (Ambion). Cells in 96-well plates (white walls, clear base; Greiner) were grown to 70 % confluency in phenol-red-free cell culture medium, and were transfected with 90 ng of replicon RNA using the TransIT–mRNA transfection kit (Mirus). Transfected cells were incubated at 37 °C in the presence of 60 µM Enduren Live Cell renilla luciferase substrate (Promega) for the times indicated, and luminescence was measured at 37 °C using a Hidex Chameleon plate reader. Chemical inhibitors were added at the time of transfection and were present throughout the assay.

### Western blotting

Huh7-Lunet T7 cell lines grown in six-well plates were lysed in lysis buffer [120 mM NaCl, 50 mM Tris, pH 7.5, 0.5 %, v/v, octylphenoxypolyethoxyethanol (IGEPAL)], and the protein content of the lysates assayed using a BCA assay kit (Pierce) using BSA standards. Equal quantities of the lysates were added to 3× SDS-PAGE sample buffer (New England BioLabs), and proteins were separated by SDS-PAGE and transferred to nitrocellulose membranes. The membranes were probed with antibodies against PI4KIIIα, PI4KIIIβ or actin.

## Supplementary Data

449 Supplementary File 1
