# Supplementary material for: Foot-and-mouth disease virus replicates independently of phosphatidylinositol 4-phosphate and type III phosphatidylinositol 4-kinases
Source: J Gen Virol. 2016 Aug;97(8):1841–52. doi: 10.1099/jgv.0.000485 (PMC5156328; doi:10.1099/jgv.0.000485)
Supplement: Supplementary file 1 [file jgv-97-1841-s001.pdf]

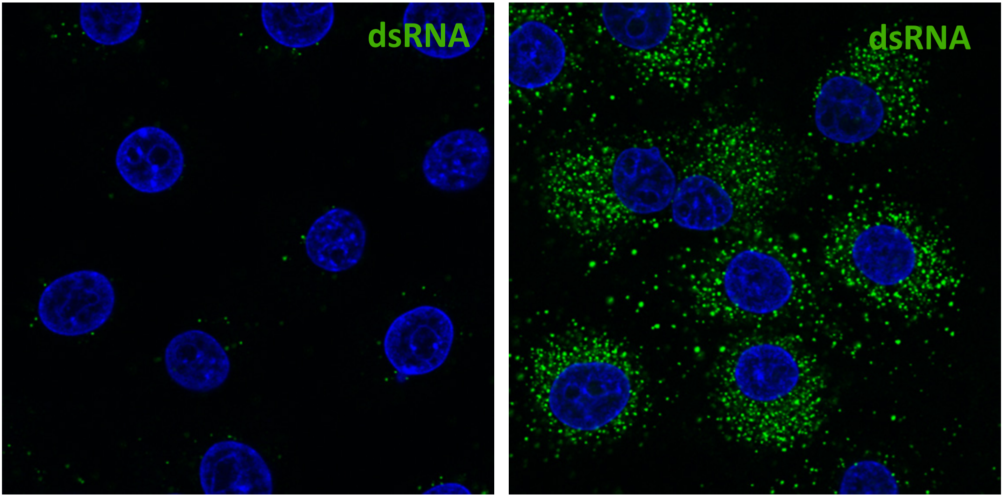

(a) Mock Infected (b) FMDV Infected

Suppl. Fig. 1: dsRNA in uninfected and FMDV-infected IBRS-2 cells.
IBRS-2 cells grown on glass coverslips were either mock infected (a) or infected with FMDV (b) for 3h45, fixed and processed for confocal microscopy. Cells were labelled with Mab J2 (English and Scientific Consulting) which detects lengths of dsRNA ≥40bp and Alexa-fluor-488 conjugated secondary antibody (green), and nuclei were labelled with DAPI (blue). There were very few dsRNA puncta in mock infected cells (a), but numerous dsRNA puncta were seen in FMDV infected cells (b).

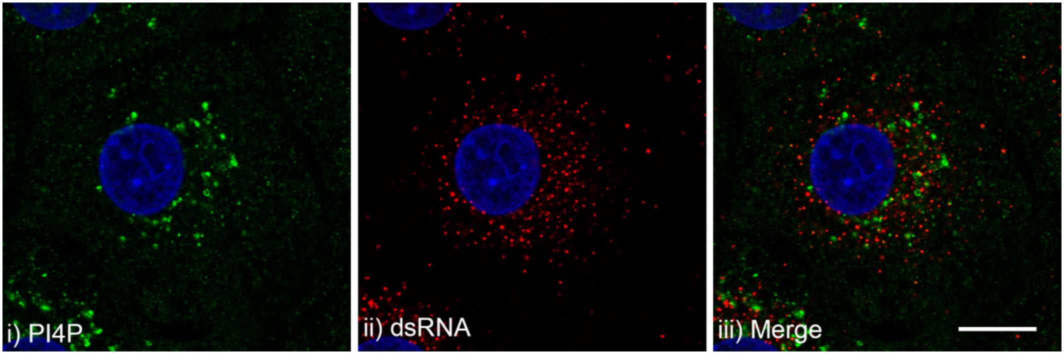

Suppl. Fig. 2: dsRNA does not show extensive colocalisation with PI4P lipid in FMDV-infected IBRS-2 cells.
IBRS-2 cells grown on glass coverslips were infected with FMDV for 3h45, fixed and processed for confocal microscopy. Cells were permeabilised with 50μg/ml digitonin and subsequently labelled with an anti-PI4P antibody to detect intracellular PI4P lipid (i – green), and co-labelled with Mab J2 to detect dsRNA (ii -red), and nuclei were labelled with DAPI (blue). Scale bar = 10μM Extensive colocalisation between PI4P and dsRNA was not seen - the majority of PI4P labelled structures were negative for dsRNA, and the majority of dsRNA puncta were negative for PI4P.
